# MRI signal intensity differentiation of brainstem encephalitis induced by Enterovirus 71: a classification approach for acute and convalescence stages

**DOI:** 10.1186/s12938-016-0136-7

**Published:** 2016-02-25

**Authors:** Hongwu Zeng, Wenxian Huang, Feiqiu Wen, Yonker Wang, Yungen Gan, Weibin Zeng, Ranran Chen, Yanxia He, Zaiyi Liu, Changhong Liang, Kelvin K. L. Wong

**Affiliations:** School of Medicine, Southern Medical University, Zhongshan Er Road No. 106, 510080 Guangzhou City, Guangdong Province People’s Republic of China; Department of Radiology, Guangdong General Hospital, Guangdong, People’s Republic of China; Radiology, Neurology and Pediatric Intensive Care Unit Department, Shenzhen Children’s Hospital, Guangdong, People’s Republic of China; Department of Radiology, College of Medicine, University of Kentucky, Lexington, KY USA; School of Medicine, Western Sydney University, Locked Bag 1797 Penrith, Campbelltown, NSW 2751 Australia

**Keywords:** Enterovirus 71, Brainstem encephalitis, Neuroimaging, MRI signal intensity, Intensity histogram

## Abstract

**Background:**

The objective of this study is to assess standardized histograms of signal intensities of T1 signal and T2 signal on sagittal view without enhancement during (1) acute stage, and (2) convalescence stage of pediatric patients with Enterovirus 71 related brainstem encephalitis (BE), and with respect to (3) healthy normal.

**Methods:**

Our subjects were hospitalized between March 2010 and October 2012, and underwent pre- and post-contrast MRI studies. The research question to be answered is whether the comparison of the MRI image intensity histograms and relevant statistical quantification can add new knowledge to the diagnosis of BE patients. So, both 25 cases in acute stage with prolonged T1 and T2 signal, without enhancement, and 13 cases in convalescence stage were introduced. In additional, a healthy group with 25 cases was recruited for comparison.

**Results:**

MRI signal intensity histogram changes of the lesions were compared at the acute and convalescence stages of the disease. Our preliminary results suggest that standardized histograms of signal intensities and their statistical properties are able to provide diagnostic information for the clinical assessment of the disease. Different stages pertaining to the histogram plots comparison showed that overall T1 signal intensity values increase as we traverse from the acute stage to the convalescence stage. And then for the healthy subjects, the T2 signal intensity values changed their magnitudes in a reverse direction. However, exceptions of this can happen in four cases where the primary lesions occurred in the brainstem that developed encephalomalacia resulting in a lower signal in T1WI and higher signal in T2WI. Statistical analysis revealed there was significant difference of T1 signal intensity among the three groups; and also, the T2 signal intensity was lower than other two groups.

**Conclusions:**

Standardized histogram of T1 and T2 intensity provide valuable and useful information for disease diagnosis and evaluation, which can potentially help medical doctors to save the lives of children.

**Electronic supplementary material:**

The online version of this article (doi:10.1186/s12938-016-0136-7) contains supplementary material, which is available to authorized users.

## Background

Brainstem encephalitis (BE) is a condition that is caused by Enterovirus 71 (EV71). EV71 is a virus that has a vital etiological role in epidemics of neurological diseases in children typically below the age of three [1–3]. It also comes with cardio-respiratory symptoms and death is imminent [4–6]. MRI qualitative studies of EV71 induced brainstem encephalitis in children have been performed in the past [7–9]. The MR intensity of diseased patients comes with certain characteristics, which include prolonged T1 and T2 signal intensity at the junction between the pons and medulla oblongata [10]. Now, the diagnostics characterization of lesions using MRI is still at its infancy, and whether the disease is permanent or transient have not yet been possible to properly diagnose. As such, a way to know how this disease deteriorates under the revelation of MRI is clinically attractive and has high medical value. It is well known that EV71 has been playing a major role in hand foot mouth disease (HFMD) or herpangina in Asia [11]. Due to the very limited literature on enhanced MRI used for the diagnosis of HFMD-related brainstem encephalitis [10], we decided to embark on a medical study of patients diagnosed with BE. This will help us to further understand HFMD and to improve its early diagnosis and to use enhanced MRI for early detection of HFMD-related brainstem encephalitis.

For an effective MRI approach, DWI/ADC maps, which were very important for the differential diagnosis and especially in making a distinction from acute infarction, can be used to facilitate the effective diagnosis of BE. The MRA or SWI methods are also existing gold standard procedures for this disease examination based on medical imaging. Where the spinal cord might be involved simultaneously [4, 7, 12], the medical scans need to include the upper and lower torso of the human. Note that BE is often combined with neurogenic pulmonary edema in which early clinical management is critical.

**Fig. 1:**
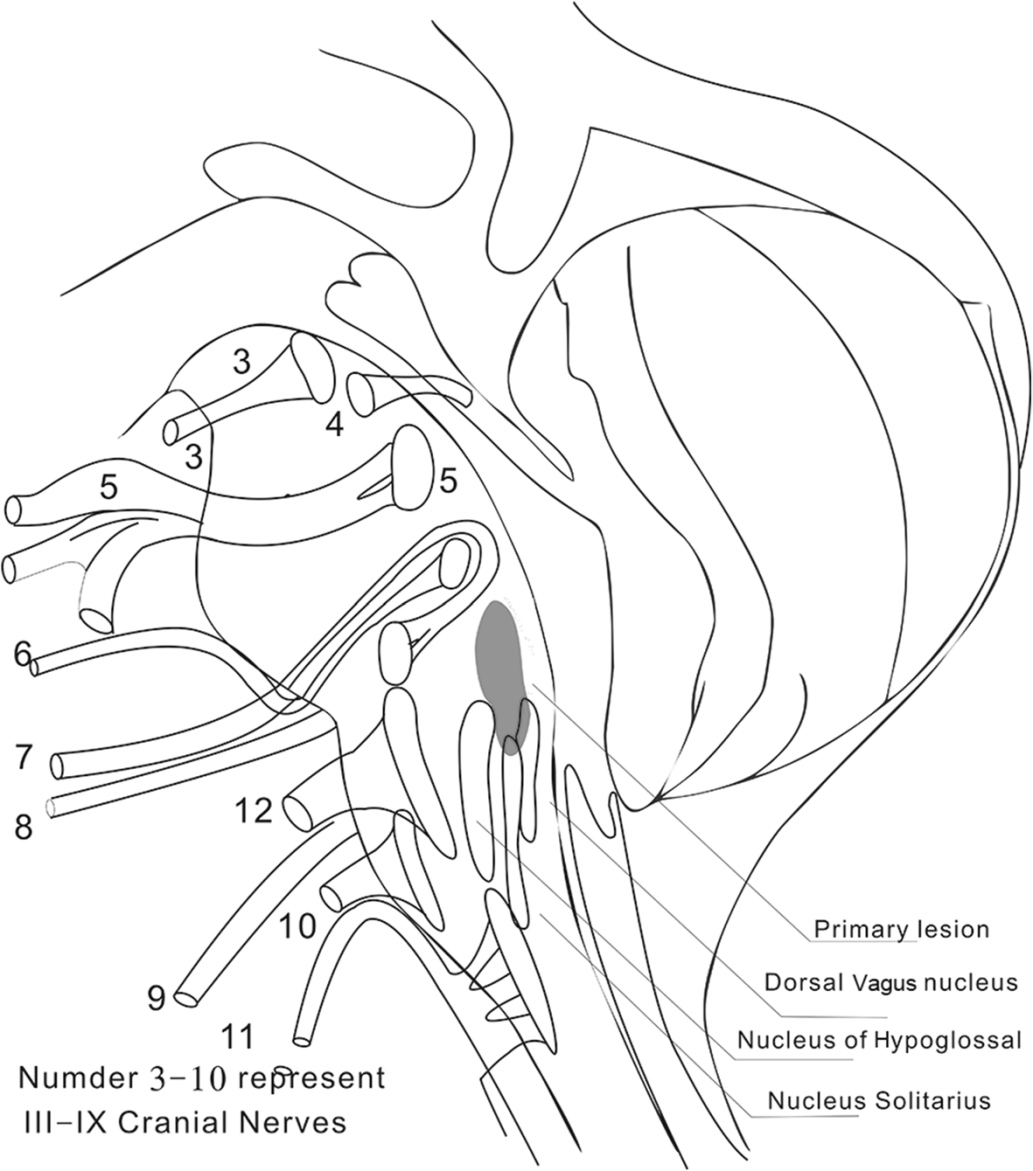
Schematic diagram depicting the location of primary lesion of brainstem encephalitis. The primary lesion is located at the posterior junction region of the pons and medulla oblongata. It is usually presented as an *oval* *shaped fleck*, which varies from 1 to 2.5 cm in length

How one can address the signal intensities modifications at various stages of the disease is the key motivation of this paper. First of all, let us provide a basic understanding of this disease. Anatomically, the primary lesion of brainstem encephalitis that is induced by EV71 is presented as a longitudinal fleck in the MR image [8, 10]. It can be located at the posterior junction region of the pons and medulla oblongata (Fig. 1; Additional files 1, 2, 3, 4, 5). The alterations of MR signal of primary lesion varied greatly due to difference course and severity of the disease [10, 12]. The MRI signal intensities of primary lesion were to be determined for both diseased and healthy groups of subjects and may be high signal intensity (defined as hyper-intensive that appears to be brighter) or low signal intensity (defined as hypo-intensive that appears to be darker). The influence of the acquisition parameters on signal intensity can be avoided by the standardization and normalization of the intensity histogram plots. For the diseased and healthy subjects, we annotated, segmented, and generated the histograms exclusively for T1WI and T2WI because of increased signal intensities.

In our study, we retrospectively reviewed 38 children having hand-foot-mouth disease and diagnosed with brainstem encephalitis. Because in southern China, there were several epidemics of HFMD between March 2010 and October 2012, and therefore, we are able to collect sufficient data for our medical image analysis. Children with severe central nervous system (CNS) complications, such as brainstem encephalitis were recruited in our study. We also enlisted 25 healthy controls into this study. Most of the deaths due to HFMD were caused by brainstem encephalitis and respiratory and circulatory failure [2, 13]. Therefore, early diagnosis is critical for the timely clinical management of the disease and to prevent fatalities [3]. This signifies the importance of establishing a medical image diagnostics framework based on MRI for helping to save the lives of children.

## Methods

### Subjects used in our study

This study was approved by the Ethics Committee of Shenzhen Children’s Hospital and informed consent was obtained from the subjects’ family member(s). MR imaging data of 38 critical pediatric hand foot mouth disease (HFMD) and/or herpangina cases complicated with brainstem encephalitis were retrospectively analyzed. These children were confirmed with EV71 infection after they were examined by a board-certified pediatric neurologist. Another 10 healthy children were recruited as control in our study.

### Medical imaging and analysis

The 38 diseased children that are diagnosed with BE underwent pre- and post-contrast cranial MRI scans. 25 cases in acute stage with prolonged T1 and T2 signal, without enhancement, and 13 cases in convalescence stage were introduced.

The first set of 25 children was scanned with MRI within the first 2 weeks. During the convalescence stage, there were another 13 children who underwent MRI scans. Finally, 25 healthy children were recruited to perform plain MRI scan. The Signa Excite 1.5 T imaging system (General Electric, Fairfield, USA) was used. Pre-contrast scan included axial T1-weighted image (T1WI), axial T2-weighted image (T2WI), axial fluid-attenuated inversion recovery (FLAIR) and diffusion weighted imaging (DWI), and sagittal T1WI. Post-contrast scan included axial and sagittal T1WI. Thickness/spacing was 6.0/2.0 mm for all axial slices, and was 3.5/0.5 mm for all sagittal slices. Sequence parameters were as follows: T1WI employed T1-FLAIR sequence (TR = 2.307 ms, TE = 10.6 ms, TI = 620 ms), T2WI used FRFSE-XL (TR = 4.000 ms and TE = 104.4 ms), FLAIR sequence parameters were TR = 8.420 ms, TE = 127 ms, and TI = 2.100 ms, EPI DWI (TR/TE 6000/82 ms, b value = 800). Post-contrast T1WI employed a FSE sequence (TR = 500 ms and TE = 20 ms). The MRI contrast agent was gadopentetate dimeglumine (Bayer, Germany). Our injection dosage was recommended to be 0.1 mmol/kg. Uncooperative patients were sedated with chloral hydrate enema (0.6 ml/kg) before the MRI scan. The first 2 weeks after the onset of the disease was clinically defined as acute stage, and after that it was set as the convalescence stage. The same procedure is performed on the 25 healthy children.

### Segmentation of brainstem encephalitis

Medflovan software, which was originally developed as a medical image visualization and analysis platform in 2007, was utilized in this study. Using Medflovan, physicians carefully performed the segmentation of the brainstem encephalitis manually for each T1WI or T2WI image slice in order to annotate the regions of interest precisely. In order to minimize the inter- and intra-observer variability, two expert radiologists were requested to perform the segmentation activity and a third party assessor then checked the two sets of segmentation contours. If there was minimal deviation and a consensus traced contours for an image, the assessor passed the segmentation as an accepted region of interest in each case. If there is a major deviation between the two sets of results, then for ease of facilitation, the assessor can utilize Medflovan to enable the correction of contour nodes and correct small details based on a suitably averaged segmentation based on the assessment of the two contours. By using the segmentation feature of Medflovan, it is easy to facilitate the differentiation and segmentation of the investigated lesion structures accurately and without bias (Additional files 1, 2, 3).

### Histograms generation method

The gray scale range of each subject’s segmented lesion image is [0–255]. A histogram based on the number of count of pixels pertaining to each intensity interval. Specifically, each interval range is determined by the entire gray scale range divided by the number of intervals designated, and serves as a bin for storing the number of pixels having intensities within this range. The numbers of pixels in all of the bins add up to the same number of pixels representing the region of interest (ROI). Segmented sizes of the lesions from the MRIs are not standardized and can vary if we extract the statistical information from the intensity histogram plot. In order to compare different intensity histograms without bias, the first step is to rescale the ROI size using a standardized number of pixel(s). As such, we are able to normalize the histograms such that the histogram entries sum up to a constant value, i.e. one pixel. Next, we standardized the number of bins used, which can be 100 in our paper. The histogram of signal intensities was generated for all images in the available data set. The smaller values on the horizontal axis represent low signal intensities (dark pixels), and the larger values represent high signal intensities (light pixels) within the ROI.

**Fig. 2:**
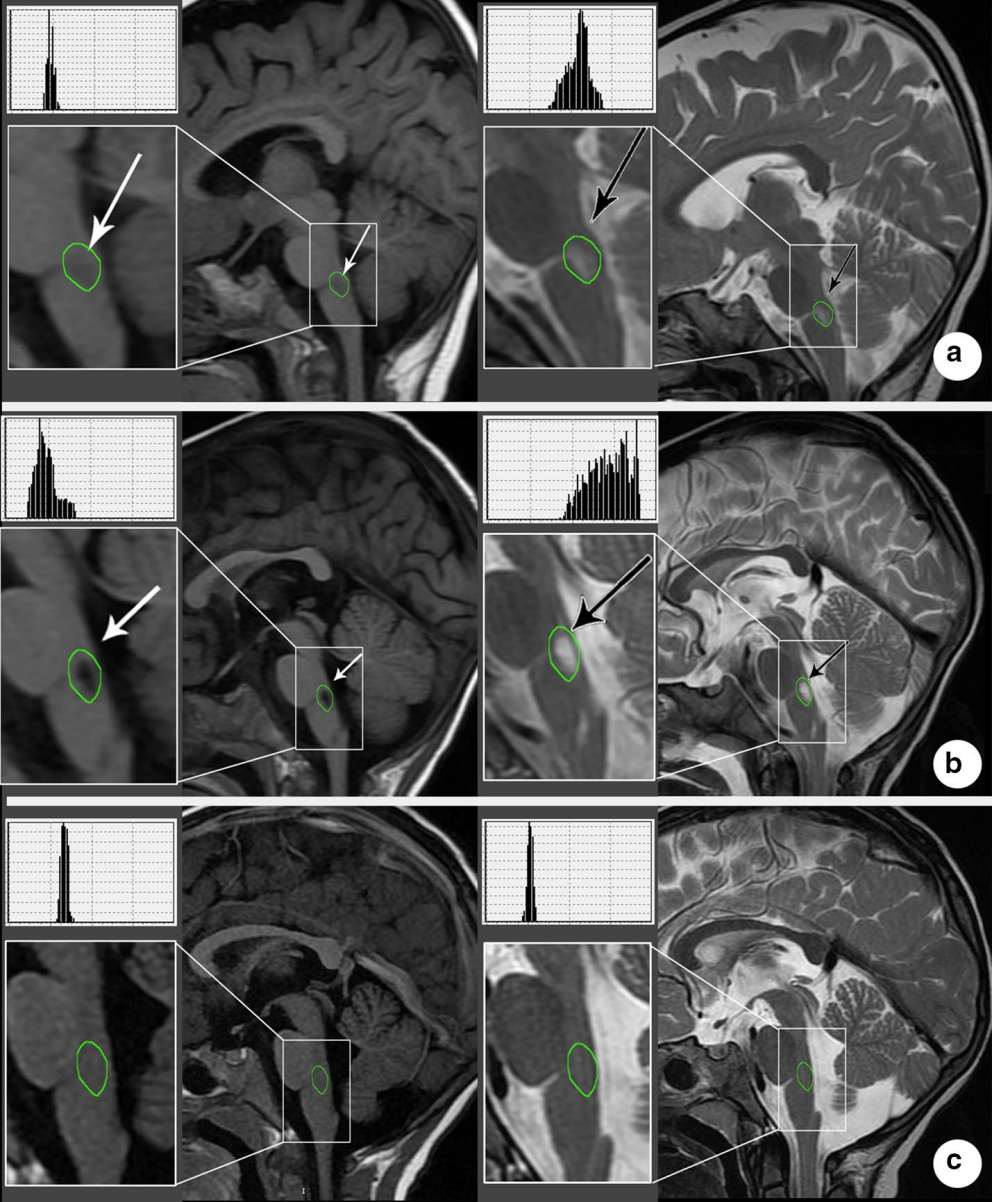
T1WI and T2WI MRI scans of diseased and healthy subjects. **a.** Acute stage: Sagittal MR images revealed a longitudinal lesion at the posterior junction region of the pons and medulla oblongata, with prolonged T1 (**a**, *white arrow*) and prolonged T2 (**b**, *black arrow*) signal. **b** Convalescence stage: MR images showed there was a longitudinal malacia cavity at the posterior junction region of the pons and medulla, with prolonged T1 (**a**, *white arrow*) and prolonged T1 (**b**, *black arrow*) signal. **c**) Healthy controls: plain MRI scan show there is no abnormality

The standardization is important as we want to compare histograms of the groups of patients at different stages of the disease. In particular, the mean and median of the intensities within the ROI (corresponding to the histogram’s center of mass) are represented by a solid and dotted lines respectively plotted within the histogram itself (see Fig. 2). Note that the standardization of number of bins and the normalization of the number of pixels has no effect on the distribution of the histogram and therefore, do not alter its mean and median. Since there was no a priori protocol to ensure that all segmented images would be geometrically constant, the standardization of histogram plots for all of the segmented lesions is important (Additional files 4, 5).

### Statistical compilation of primary lesion

The mean and median intensity values for primary lesion, which is located in the junction region between pons and medulla oblongata, are determined. Similarly, these values are measured based on the same region in non-diseased controls. The differences in scores were to be determined statistically. (P > 0.01) signifies differentiation of the two groups of subjects and accurate diagnosis of BE.

Statistical analysis was performed using SPSS version 21.0 (IBM, Chicago, Illinois). Due to the relative small sample size (*n* = 63). Normal distribution could not be assumed and therefore non-parametric statistical tests were performed. The one-way analysis of variance (ANOVA) was applied to investigate the difference in mean of intensity histogram between subjects with BE (at acute and convalescence stages, patients) and subjects without BE (healthy controls). P value less than 0.05 was considered statistically significant.

## Results and discussion

### MRI findings and classification

The primary lesion of brainstem encephalitis that is induced by EV71 was a longitudinal fleck, which located at the posterior junction region of the pons and medulla oblongata (Fig. 1). The alterations of MR signal of primary lesion varied greatly due to difference course and severity of the disease. The MRI signal intensities of primary lesion were to be determined for both acute stage, and convalescence stage of the diseased subjects, and healthy groups of subjects.

### Examination of diseased and healthy subjects using intensity histogram plots

The patterns for the acute stage and convalescence stage patients, as well as for healthy subjects were presented as a combined histograms plot based on their T1WI and T2WI as follows:

*Acute stage* The primary lesion signal alterations consisted of an interesting pattern, which pertains to prolonged T1 and T2 signal, (*n* = 25). Specifically, we observed fleck image signals that were hypo-intensive on T1WI and hyper-intensive on T2WI. There is no intensity enhancement in the post-contrast T1WI. Samples of these MR images are presented for each case (Fig. 2a).

*Convalescence stage* The primary lesion of cases displayed as prolonged T1 and T2 signal (*n* = 13). Similar to the acute stage, we observed fleck image signals that were hypo-intensive on T1WI and hyper-intensive on T2WI, and with no intensity enhancement in the post-contrast T1WI. We presented samples of their MR images and analysis (Fig. 2b).

*Healthy subjects* There were no abnormalities founded in T1WI and T2WI MRI scan (Fig. 2c).

**Fig. 3:**
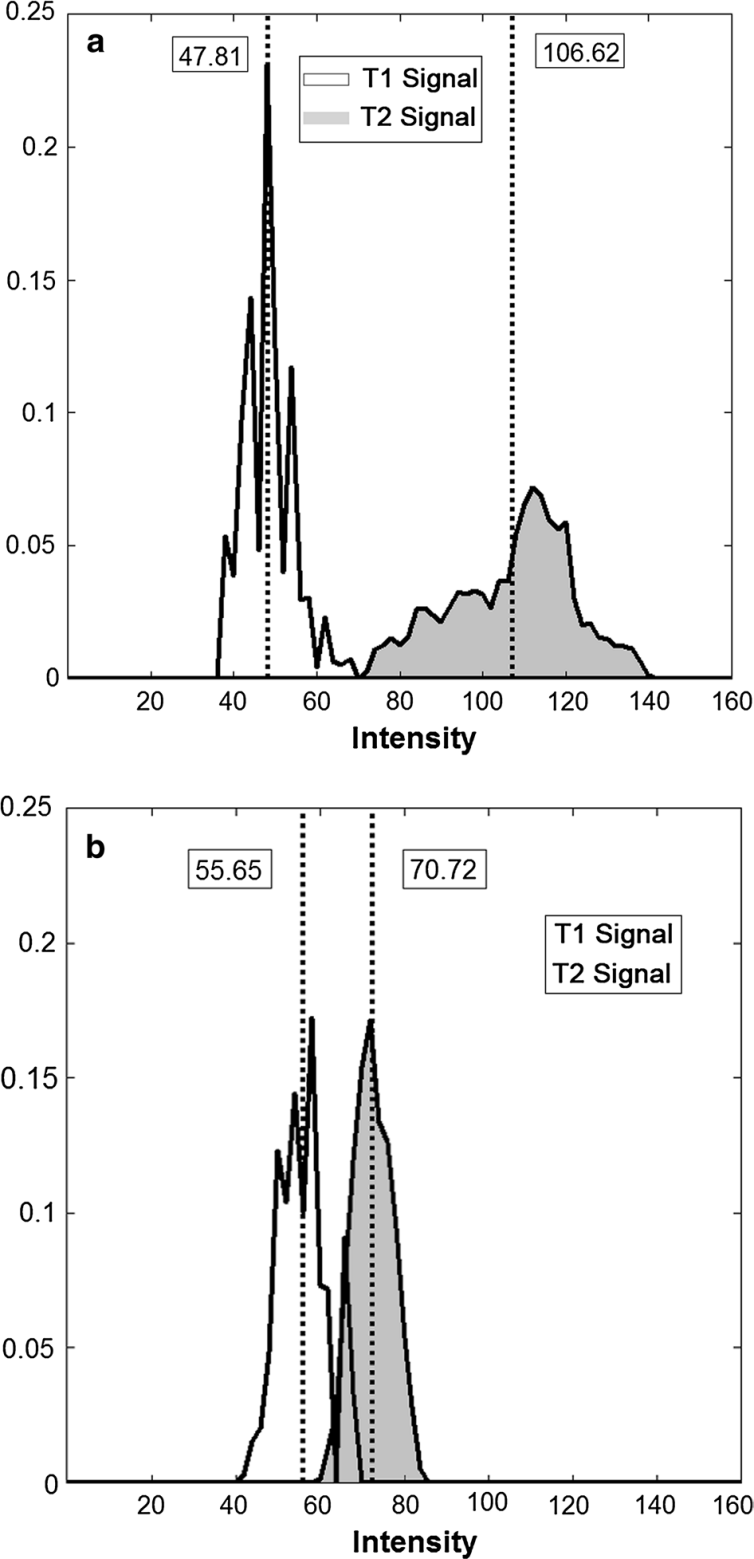
Simultaneous T1WI and T2WI histogram plots comparison based on different stages individually. **a** Histograms for T1 and T2 signal intensity at acute stage. **b** Histograms for T1 and T2 signal intensity at convalescence stage. Standardized histogram showed a lower T1 signal intensity (with the mean value of intensities at 47.81) at acute stage while compared with convalescence stage (with the mean value of intensities at 55.65). Also standardized histogram showed a higher T2 signal intensity (with the mean value of intensities at 106.62) at acute stage in comparison with convalescence stage (with the mean value of intensities at 70.72)

**Fig. 4:**
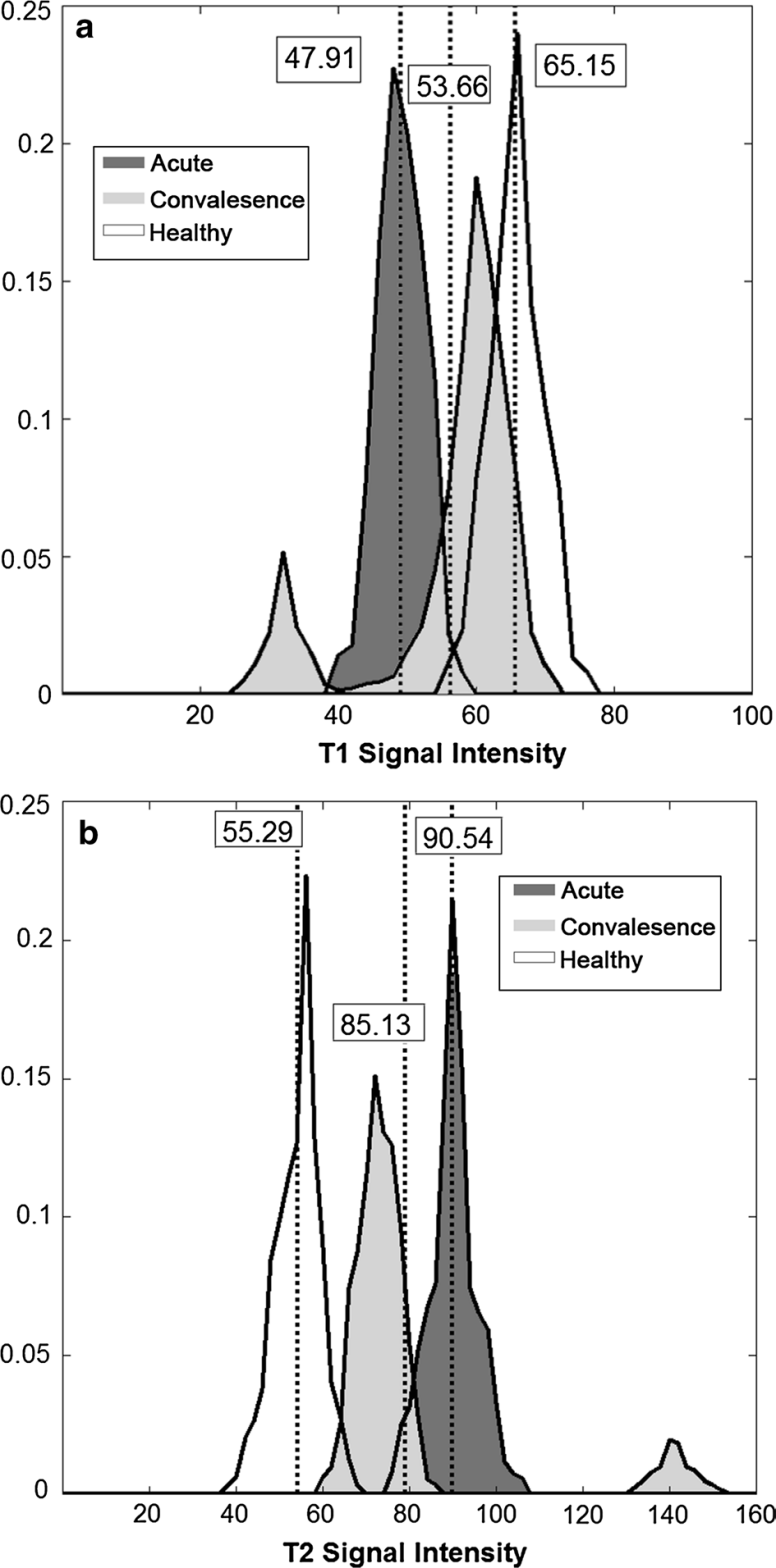
Different stages histogram plots comparison for T1WI and T2WI individually. **a** Histograms for T1 signal intensity of three groups. **b** Histograms for T2 signal intensity of three groups. In practice, the overall T1 signal intensity values increase as we traverse from the acute to the convalescence stages, and then to the healthy subjects. However, exceptions of this can happen in 4 of the cases where the condition deteriorate, the primary lesion in the brainstem that developed in conjunction to encephalomalacia resulting in a lower signal in T1WI and higher signal in T2WI. This produces an outlier represented by an extra secondary peak on the left side of the histogram (lower signal intensity values) in the T1WI, and on right side of the histogram (higher signal intensity values) in the T2WI

Traditionally, radiologists and clinical experts can only observe MRI images for an accurate assessment of the disease. However, a better approach would have been to quantify the differences in the states of MRI intensity using a more scientific approach, which is the motivation for our paper. Upon understanding that there are modifications to the intensities for the three different groups of subjects, our aim was to identify and to interpret changes of standardized histograms of T1WI and T2WI for various stages of the disease and to compare these results to establish a reliable classification system as shown in Fig. 3. For the individual set of T1WI and T2WI results, we presented the histogram plots and their distribution mean values for the three groups of subjects on a series of single graphs as shown in Fig. 4.

### T1WI and T2WI histogram plots comparison at different stages individually

In terms of just the acute stage patient, the convalescence, and the healthy subjects separately, we plot the T1 and T2 intensity histograms on the same graph. It can be observed that there is a distinct difference in the T1 and T2 intensity means of the histograms for both the acute (Fig. 3a) and convalescence patients (Fig. 3b). The simultaneous plot of T1 and T2 intensity distribution can provide us with a unique signature identifying the disease progression. For instance, if we see an overlap of the T1 and T2 distribution, it means that the patient has recovered and is healthy. Increasing difference in the mean of the two histograms signifies an aggravation of the disease. At the acute stage, the centers of their distributions become most apart. These types of histogram plot signatures can be used to effectively assess the severity of the disease condition.

### Different stages histogram plots comparison for T1WI and T2WI individually

Now, it will be interesting to know how each of the T1 and T2 intensity distributions varies across the different stages of the disease. Let us plot the T1WI intensity histogram of different stages on the same graph. We can observe clearly a gradual progression of intensity means from the acute to the convalescence stage, and to the healthy stage (Fig. 4a). This signifies that the signal intensity values are generally increasing with the recovery of the patient. Likewise, we performed the same plots for the T2WI results. On the contrary, although the signal intensities also increase from the acute to the convalescence stage, the intensity means of the healthy controls drops to even below that of the acute stage patients (Fig. 4b).

### Statistical analysis of signal intensity histogram properties

**Fig. 5:**
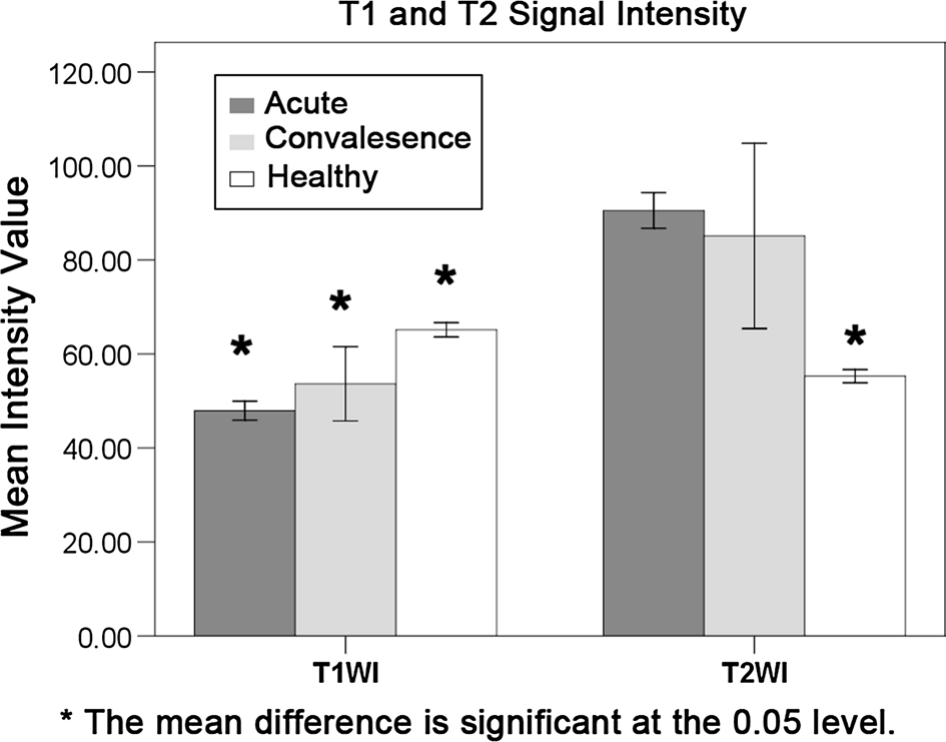
Comparison of means of T1WI and T2WI distributions for different stages of the disease for patients, and for healthy subjects. Statistical analysis revealed that there was significant difference of T1 signal intensity among the three groups; and also T2 signal intensity was much lower for the healthy subjects in comparison with the other two groups

The intensity histogram properties, i.e. mean of distribution, were presented in the following statistical bar chart using Fig. 5. It can be observed that the means of histogram plots for the BE diseased group are typically higher than their controls. This signifies there is an intensity difference in MR images of diseased patients with BE and also with the healthy subjects (P > 0.01). Therefore, differentiation of patients based on intensity analysis can be achieved statistically.

The spread of distribution histogram plots comparison for T1WI and T2WI shows some interesting patterns. We note that there is a very large standard deviation in the mean of intensity distribution for the convalescence stage patients in comparison to the acute stage patients. This may also be accompanied by the larger spread of distribution and hence, bigger area under the graph for the intensity histogram (Figs. 3, 4). This deviation is noted to be the smallest for the healthy controls. This phenomenon occurs for both the T1WI and T2WI. It can be explained by the fact that patients undergoing through the convalescence stage are recovering at different speeds and some may have reached towards the end of recovery and approximating to the intensities presented by a healthy subject. This is a very important information, which can be used to diagnose the rate of recovery of BE patients. Here, a greater hyper-intensive signal for T1WI and lower hypo-intensive signal for T2WI signifies a better recovery.

## Limitations

Firstly, the patient and healthy control numbers that we have used may be small for a normal distribution statistical analysis for the standard population study. Nevertheless, it is sufficient for our test of concept procedure. Secondly, we have not yet performed the classification system for various sub-stages or classes of the single acute and convalescence stages, which is very important in an effective diagnosis of the actual condition of the children, but this has been well-planned in our next stage of research. Thirdly, the intensity range tuning by radiologists may vary due to different perceptions of the appropriate lesion definition and intensity differentiation, and therefore affect the means of the T1W1 and T2WI histograms for the comparison at different stages of disease. This may be rectified by a consistent intensity range for all of the images examined prior to their extraction and intensity histogram plotting. However, this may not allow enough intensity differentiation for the lesion to be visualized. In fact, our proposed technique may be more suitable for CT images since their intensity range is defined to be constant, and lesions are well defined by different image intensities.

## Conclusion

The patients in their critical condition of being affected by EV71 suffer from neurological issues such as aseptic meningitis, acute flaccid paralysis, and brainstem encephalitis. Brainstem encephalitis can result in neurogenic pulmonary edema/hemorrhage and cardiorespiratory failure, or even fatality. The neurological manifestations of brainstem encephalitis induced by EV 71 may include myoclonus and tremor and therefore, a useful diagnostics system is urgently required to help save lives. The signal intensity of T2WI can help to distinguish brainstem encephalitis patients having hyperintense lesions, lesions with isointensity or hypointensity. The contrast enhancement based on pre- and post-contrast can identify the presence of the lesion. Above all, the unique signature generated by the configuration of intensities for both the T1WI and T2WI can be used to assess the recovery success of the patient; And to the best of our knowledge, we are the first in the world to establish a quantifiable technique for monitoring the severity of the disease. This paper has introduced a robust and reliable technique for detecting the severity levels of BE disease accurately and non-invasively. This presented method and its underlying concept may be used to develop a clinically attractive medical image diagnostic system in the long run.

## Additional files

10.1186/s12938-016-0136-7 Sagittal MR image demonstrates the lesion of brainstem encephalitis induced by EV71.
10.1186/s12938-016-0136-7 To traced contours of the brainstem encephalitis lesion for segmentation.
10.1186/s12938-016-0136-7 After tick the segment region, Medflovan show the segmentation region of lesion.
10.1186/s12938-016-0136-7 Medflovan displays signal intensity histogram.
10.1186/s12938-016-0136-7 Shows the graphic display option and statistic number.
