# Supplementary figures and images for: MRI signal intensity differentiation of brainstem encephalitis induced by Enterovirus 71: a classification approach for acute and convalescence stages
Source: Biomed Eng Online. 2016 Feb 25;15:25. doi: 10.1186/s12938-016-0136-7 (PMC4766649; doi:10.1186/s12938-016-0136-7)

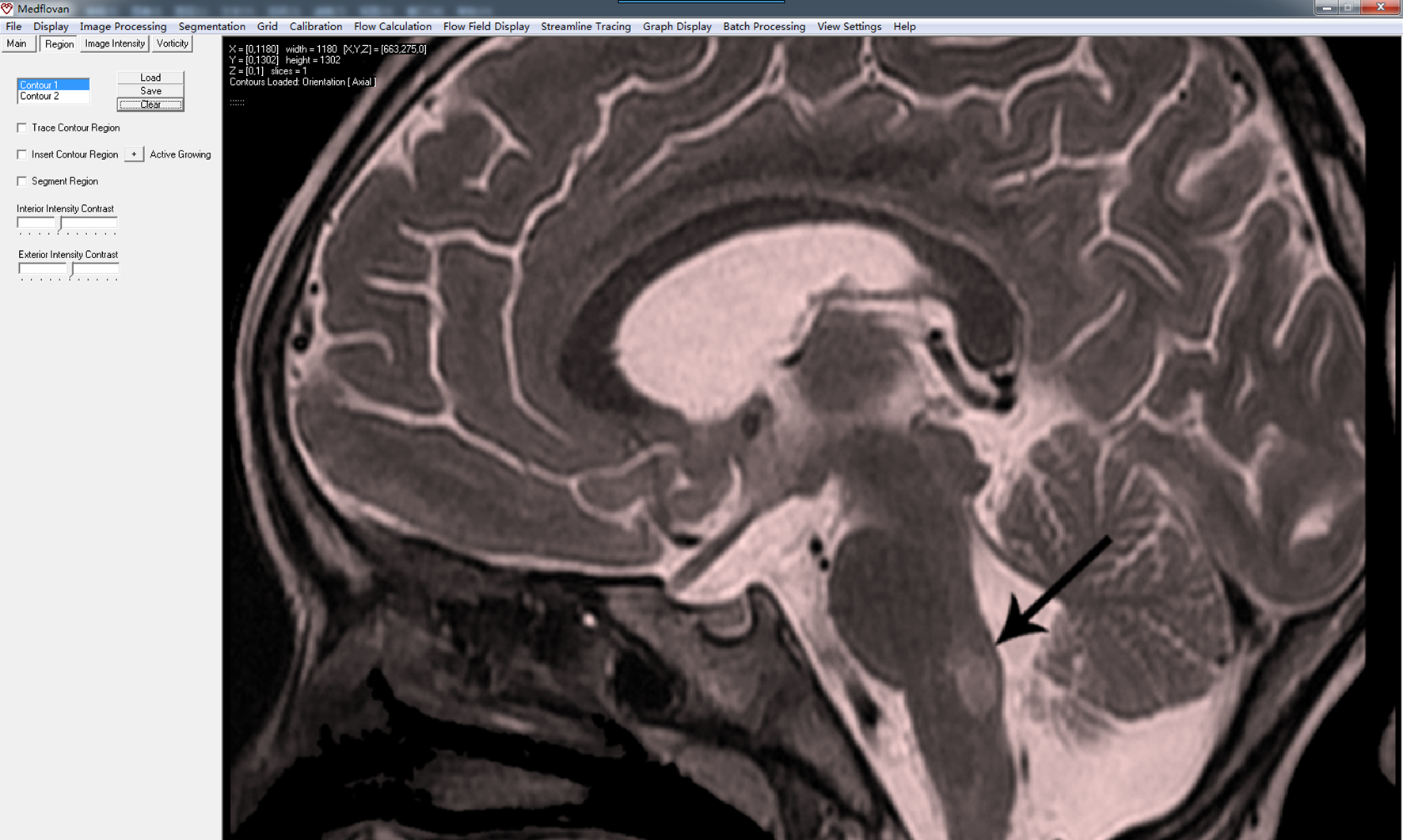

Supplement: Supplementary file 1 — 10.1186/s12938-016-0136-7 Sagittal MR image demonstrates the lesion of brainstem encephalitis induced by EV71. [file 12938_2016_136_MOESM1_ESM.tif]

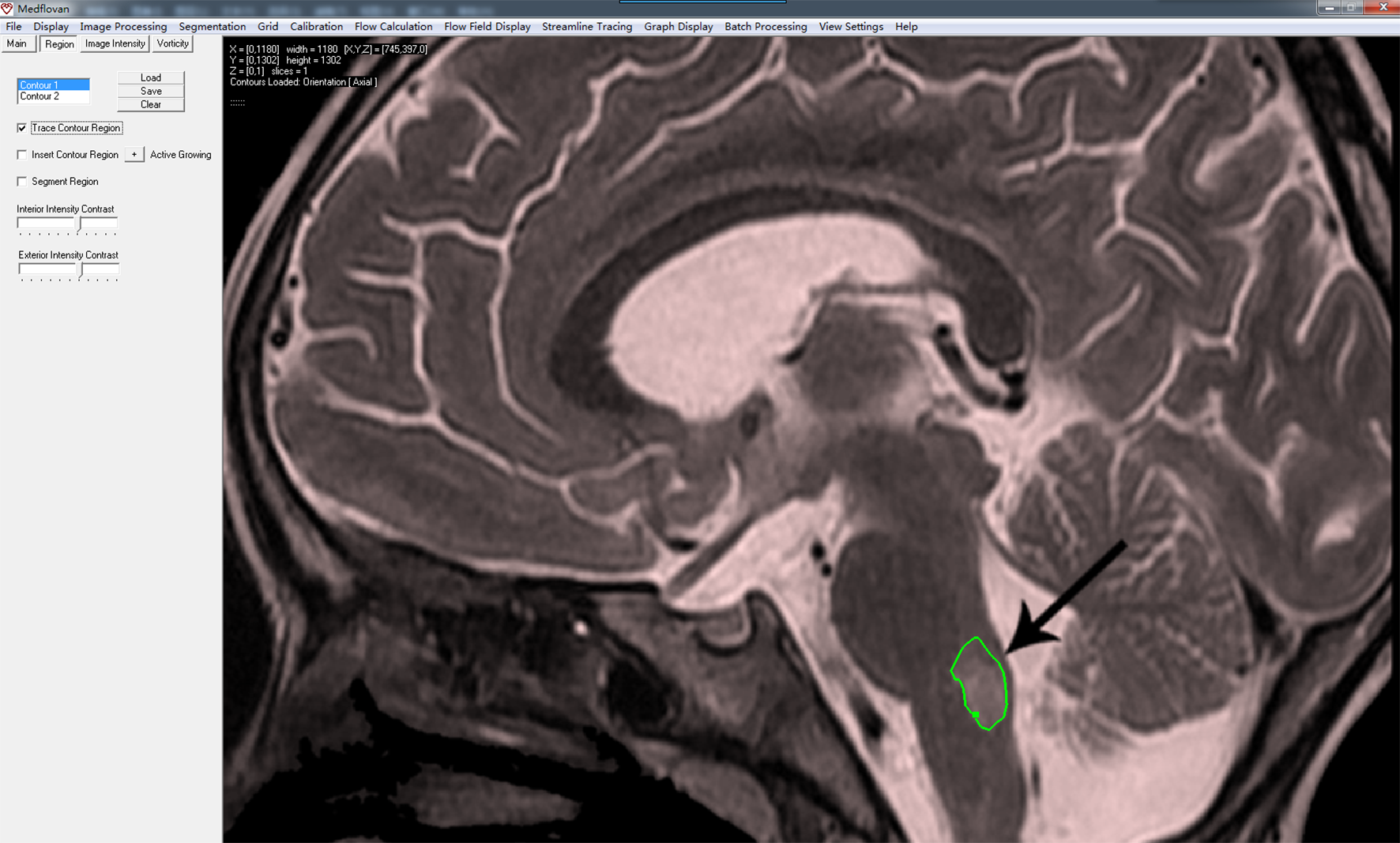

Supplement: Supplementary file 2 — 10.1186/s12938-016-0136-7 To traced contours of the brainstem encephalitis lesion for segmentation. [file 12938_2016_136_MOESM2_ESM.tif]

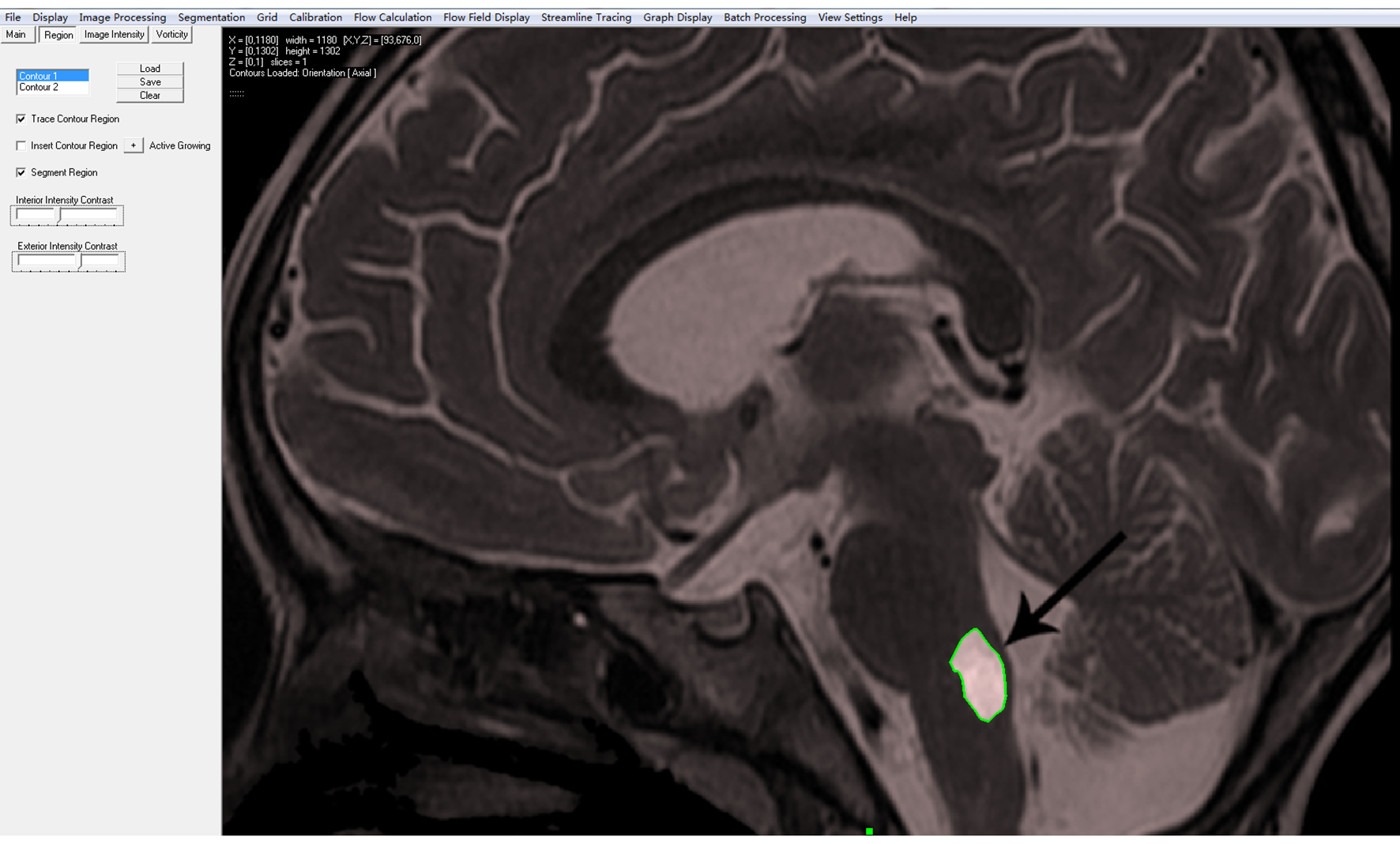

Supplement: Supplementary file 3 — 10.1186/s12938-016-0136-7 After tick the segment region, Medflovan show the segmentation region of lesion. [file 12938_2016_136_MOESM3_ESM.tif]

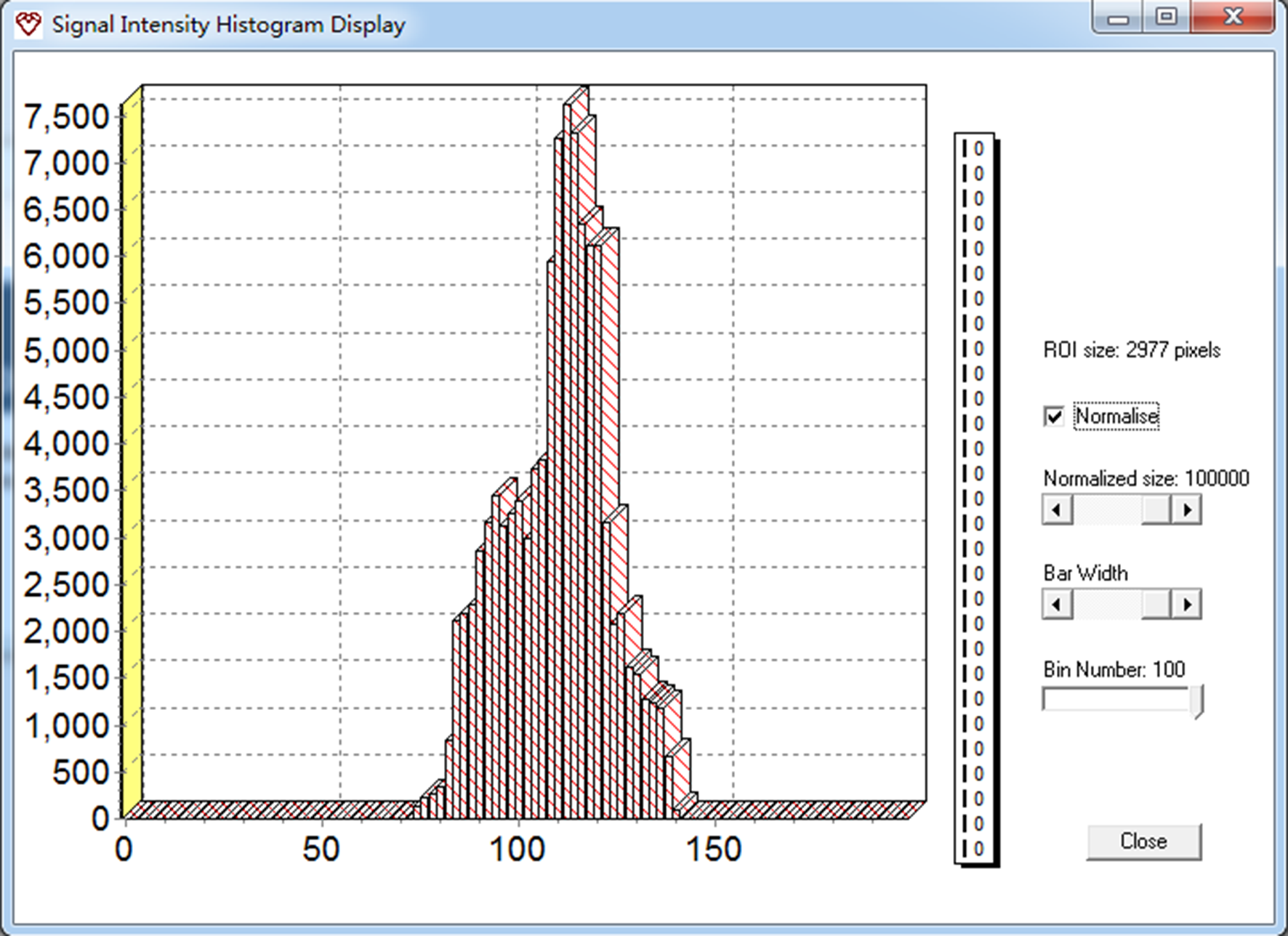

Supplement: Supplementary file 4 — 10.1186/s12938-016-0136-7 Medflovan displays signal intensity histogram. [file 12938_2016_136_MOESM4_ESM.tif]

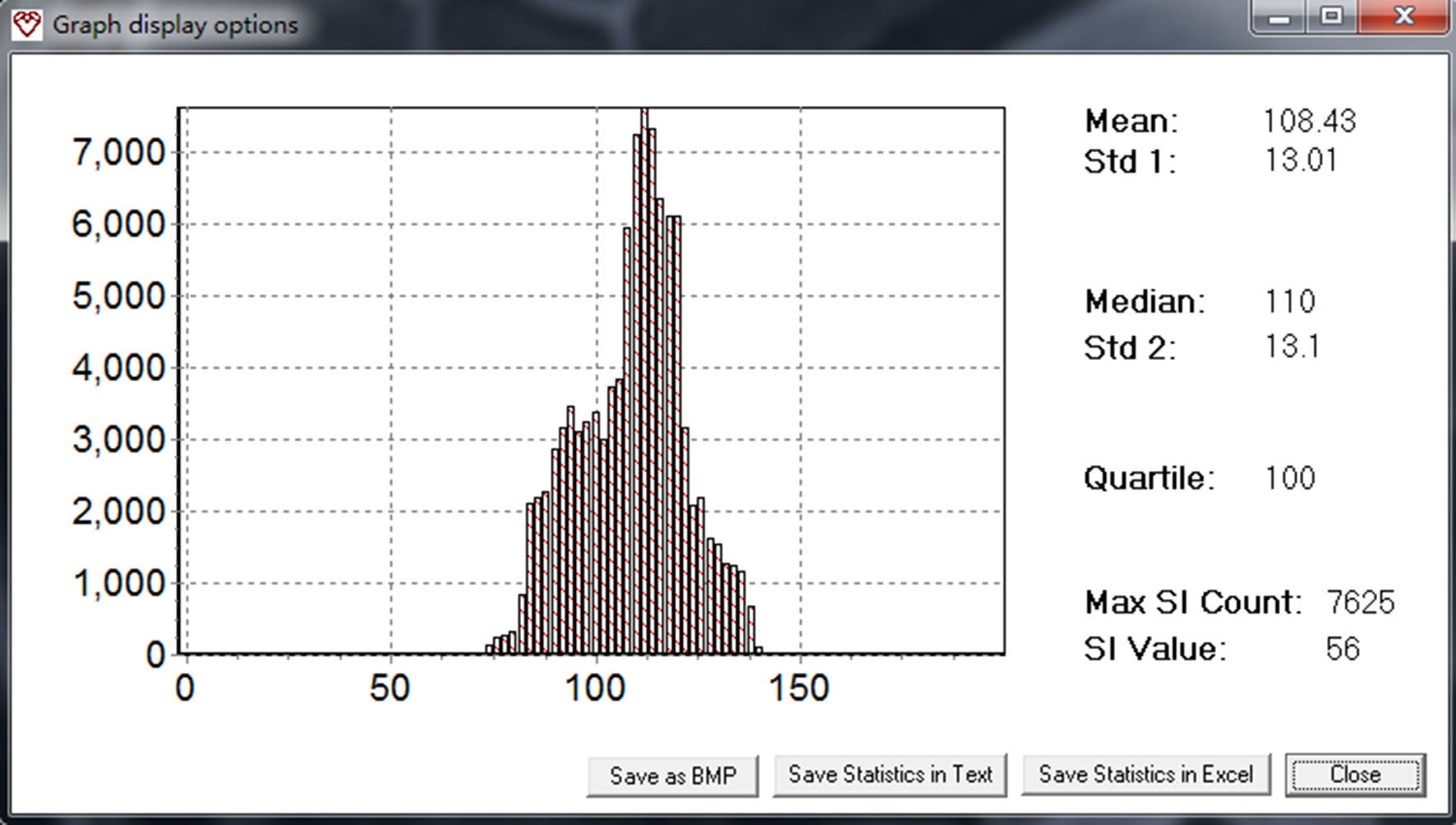

Supplement: Supplementary file 5 — 10.1186/s12938-016-0136-7 Shows the graphic display option and statistic number. [file 12938_2016_136_MOESM5_ESM.tif]
